# A review on adult pragmatic assessments

**Published:** 2014-07-04

**Authors:** Davood Sobhani Rad

**Affiliations:** ^1^ Department of Speech Therapy, School of Rehabilitation, Tehran University of Medical Sciences, Tehran, Iran

**Keywords:** Adult Pragmatic, Communication, Functional Assessments, Discourse Analysis, Assessment Tools, Right Hemisphere

## Abstract

Pragmatics is defined as appropriate use of language either to comprehend ideas or to interact in social situations effectively. Pragmatic competence, which is processed in the right hemisphere, comprises a number of interrelated skills that manifest in a range of adaptive behaviors. Due to the widespread influence of language in communication, studying pragmatic profiles, by developing appropriate tools, is of importance. Here, a range of pragmatic theories and assessment instruments available for use in adult patients is reviewed.

## Introduction

Pragmatic, which links linguistic knowledge to communicative proficiencies, is an appropriate use of language across a variety of social contexts that provides accurate interpretation of intentions and references.^1^ Three types of knowledge have been introduced as prerequisites for appropriate communication in context; linguistic knowledge including syntax, semantic and phenology; knowledge of objects, events, and actions, and social knowledge that governs conversation and behavior in the society.^2^ Pragmatic competence is a real-time behavior; therefore, communication adjustments must rely on simultaneous processing of the environment during the communicative act. This processing deploys a number of resources such as attention, feedback, and executive functioning. During the last decade, it has been indicated that communicative skills are processed in the right hemisphere; this is the reason why pragmatic impairment is very common in patients with right hemisphere damage.

Loss of pragmatic communication skills, known as apragmatism or pragmatic aphasia, impairs individual’s ability to effectively convey his needs, and to elicit help from others. Whereas the detection of language form problems is relatively straightforward, pragmatic language problems are more difficult to detect since language pragmatism is dependent on the specific context and implicit rules. From the clinical point of view, essence of pragmatic assessment and therapy is to capture and measure different components of pragmatic competence, and, if possible, to enhance patient’s ability to adapt to a changing communicative environment.^2^ Given the complexity of pragmatic language behaviors, assessment of pragmatics can be difficult, leaving many clinicians to rely on non-standardized, observational methods that can be challenging for determining service eligibility.^3^^,^^4^ In this review, a number of adult pragmatic theories and assessment instruments will be introduced.

**Pragmatic Theories**

Since sensitivity to task and context is the core of pragmatic competence, precise, and accurate assessments of pragmatic abilities are depend on multidimensional evaluation. Characterization of pragmatic competence by various groups has led to the development of a number of evaluation measures that have in common their basis in a theoretical framework and a multidimensional perspective of both linguistic and nonlinguistic elements.^5^ Here, a number of common and important pragmatic theories will be reviewed briefly.

***Speech act theory***

Speech act theory^6^^,^^7^ focuses on the communicative functions of utterances, and attempts to explain the use of language to accomplish intended actions and its resulting effects on the addressee. Five categories of speech acts were identified based on the functions assigned to them including representatives, directives, expressives, comissives, and declaratives.^8^

Despite speech act theory has had a tremendous influence on functional aspects of pragmatic theory; it has also received very strong criticism. For instance, many scholars including Langshaw Austin^6^ and Searle^7^based their work principally on their intuitions and focused exclusively on single, isolated utterances independently of discourse context. However, speech act involves a communicative function and cannot take the form of a sentence, which is only a grammatical unit within the system of language.^9^ A further assumption commonly made by speech act theorists is the passive role of the hearer, by which interactional aspects are neglected. Speech act theory, in that it does not consider the function played by utterances in driving conversation is, therefore, insufficient in accounting for what actually happens in conversation.^10^

***Conversational implicature***

Theory of conversational implicature^11^ proposes that all conversants follow a cooperative principle that determines the way in which language is used with maximum efficiency to achieve rational communication. A conversational maxim is any of four rules stating that a speaker is assumed to make a contribution that is adequately informative (quantity maxim), truthful (quality maxim), relevant (relation maxim), and orderly (manner maxim).

Similar to speech act theory, despite its wide use in experimental studies, Grice’s theory of conversational implicature is not always easy to apply in the analysis of clinical data. As in clinical criteria for pragmatics that distinguish implicatures, a number of maxims might be missed, judgments are invariably made from the perspective of a speech pathologist who makes implicit comparisons with normal conversational behavior.^12^

***Relevance theory***

Relevance theory is a framework to study cognition by providing a psychologically realistic account of communication. This theory has been introduced as an inferential approach to pragmatics that explains how the hearer infers the speaker’s meaning on the basis of provided evidence.^13^ Relevance theory has had considerable influence in the disputed borderlands between semantics, pragmatics and philosophy of language, including debates about the extent to which pragmatic inference affects the proposition expressed by an utterance.^14^^,^^15^ Nevertheless, since the theory models communication from the perspective of the hearer, critics claim that it fails to take sufficient account of the collaborative and reciprocal nature of communication between individuals.^16^

***Discourse analysis***

Discourse analysis is the study of language use beyond the sentence boundary and focuses on higher units that are coherent sequences of sentences, propositions, speech acts and conversation turns. In clinical linguistics, discourse analysis is a conversational sampling procedure oriented toward functional aspects of social action at the descriptive level of analysis. In spite of being designed to discover potential conversational difficulties, discourse analysis cannot determine, by itself, the presence or absence of a language disorder.^17^

***Conversation analysis***

Although conversation analysis may be regarded as a type of discourse analysis,^18^ it is also being considered separately due to its application in assessment of communication disorders. The distinctness of conversation analysis from other approaches to discourse analysis is its emphasis on the conversation as an integral feature of social interaction. It views conversation between participants and examines the significance of sequential phenomena, and the way in which participants orient to each other and manage the interaction generally.^19^ Moreover, conversation is known as continuously shaping and renewing the context; features such as turn organization,^20^ conversational repair,^21^ speaker overlap,^22^ repetition,^23^ and prosody^24^ are considered crucial in conversational analysis.

Despite “pragmatic impairment” refers to cognitive abnormal behaviors reported in a wide range of neurologic disorders, it lacks discrimination and is hardly adequate as a diagnostic descriptor.^12^ In this regard, it is essential that neurolinguists and clinicians define a more comprehensive, semiotic view of pragmatics since the phenomenon of pragmatic disability is not adequately accounted for by at least some mainstream pragmatic theories.

As it was reviewed above, theories of pragmatics provide reasonable means of describing pragmatic impairments, however, the level of explanation they afford is rarely helpful to clinicians, in that they do not translate easily into clinical intervention. A holistic approach to pragmatics, which takes account of the behavior and its contributed underlying factors, helps clinicians to better understand and, therefore, treat pragmatic impairments and also attracts more attention on features of communicative interactions that are not adequately considered by current theories.^12^

**Pragmatic Assessment Tools**

Several pragmatic approaches, with increasing clinical popularity, have been introduced for adult pragmatic assessment. However, traditional language assessment models encompassing phonetic, syntactic and semantic aspects were not sufficient to determine the impact of patients’ disabilities on their everyday life, since patients who presented little or no deficit on formal language tests had substantial problems in the social use of communication. Accordingly, there was a need to design theoretically grounded, wide-range instruments capable of assessing various kinds of pragmatic phenomena by different means, such as linguistic, extra-linguistic, and paralinguistic communication.

To define profiles of impairment, pragmatic assessments identify and measure single cognitive processes underlying a range of communication behaviors. As mentioned before, pragmatic assessments mostly differ in two dimensions; the extent to which they are based on the underlying theory, and the components of pragmatic competence which they tap.

A number of pragmatic assessment tools examine specific splinter components of pragmatic competence, for instance, topic coherence analysis,^25^ discourse comprehension test (DCT),^26^ and comprehension of inferred meaning test.^27^ However, other measures are more inclusive and incorporate a wide range of behaviors. These include pragmatic protocol (PP),^28^ profile of communicative appropriateness (PCA),^29^ assessment protocol of pragmatic-linguistic skills,^30^ discourse abilities profile,^31^ revised version of Edinburgh functional communication profile (FCP),^32^ communicative abilities in daily living (CADL),^33^^,^^34^ communication competence self-report,^35^ expression, reception and recall of narrative instrument,^36^ and verbal pragmatic rating scale.^37^

Although profiling specific pragmatic strengths and weaknesses leads to identification of processes involved in communication, it could not outline the consequences of the communication deficit in individuals’ daily interactions. For such evaluation, functional assessments can be applied to measure a person’s ability to communicate efficiently in real life situations without directly identifying the componential abilities underlying communication. Examples of such tests are FCP^38^ and functional assessment of communication skills (FACS) for adults.^39^

Among all the introduced instruments, most commonly-used checklists designed for adult pragmatic and functional assessments are DCT, PP, PCA, CADL, FCP, and FACS (Table 1). To have a better understanding and evaluation, mentioned tools are explained in more details in the following.

***DCT***

DCT^26^ is a well-controlled measure of narrative processing that has good psychometric properties, and also taps comprehension of implied information as well as explicitly-conveyed information. DCT permits ensitive and reliable measurement of changes in both listening and reading comprehension of discourse over time, and provides information useful for planning treatment and for counseling communication partners. However, as mentioned earlier, DCT is a uni-dimentional assessment tool, and therefore, is not recommended for a wide range pragmatic analysis.

***PP***

PP,^40^ which is based on speech act theory is a general observation profile on which 30 communicative abilities are rated in a two-point scale. All pragmatic parameters in PP checklist are assigned to three aspects: (i) verbal behaviors such as speech acts, message specificity, cohesion and topic selection, initiation and maintenance; (ii) paralinguistic behaviors including fluency, prosody, vocal quality, and speech intelligibility; and (iii) non-verbal behaviors such as facial expressions, eye gaze, and gestures. Although PP might provide important information on communicative abilities, this possible advantage is at the cost of psychometric limitations. For instance, very heterogeneous variables of PP are only scored as appropriate or inappropriate, while no clear criteria is presented for these two scales. Furthermore, it has been argued that the distinction between pragmatic behaviors and language impairments is less cleared than suggested by authors.^41^

***PCA***

Penn^29^ framed the conceptual base for PCA in speech act theory and follows closely the analysis of pragmatics proposed in Levinson.^5^ This profile chart consists 45 parameters that are grouped in six main sections; response to interlocutor, control of semantic content, cohesion-fluency, sociolinguistic sensitivity, and non-verbal communication. PCA requires a marking system using a five-point scale that, in comparison with PP, provides a more sensitive analysis; however, it adds difficulty in inter-scorer reliability.^42^

***CADL***

CADL^33^^,^^34^ is another pragmatic instrument based on speech act theory that makes use of role-playing, and as a result, reproduces everyday social situations. This assessment contains 68 items and uses a heterogeneous series of communicative interactions that are scored for functional appropriateness on a three-point scale. The number of messages measures communicative attitude, and the comprehensibility of the messages measures communicative efficiency.

As a functional assessment test, CADL is suitable for test–retest assessments due to its high level of scoring reproducibility. Moreover, it includes the possibility of plotting and analyzing the component abilities tapped by each task, and this enhances the test’s usefulness as a therapy planning tool.^43^

**Table 1:** Most commonly used instruments for adult pragmatic and functional assessments

**Assessment tools**	**Component of pragmatic test**	**Scale**	**Reports**
Discourse comprehension test (DCT)^26^	Two sets of five stories	Two-point scale Yes No	47-55
The pragmatic protocol (PP)^40^	Verbal aspects	Two-point scale Appropriate Inappropriate	28,42,56-64
	Paralinguistic aspects		
	Non-verbal aspects		
Profile of communicative appropriateness (PCA)^29^	Response to interlocutor	Five-point scal Inappropriate Mostly inappropriate Some appropriate Mostly appropriate Appropriate	42,65
	Control of semantic content		
	Cohesion, fluency		
	Sociolinguistic sensitivity		
	Non-verbal communication		
Communicative abilities in daily living (CADL)^33^^,^^34^	Speech act	Three-point scale Correct Adequate Wrong	66-70
	Utilizing context		
	Social convention		
	Sequential relationships		
	Read, write and calculate		
	Deixis		
	Role-playing		
	Non-verbal symbols		
	Divergent thinking		
	Humor, absurdity, and metaphor		
Functional communication profile (FCP)^38^	Movement	Nine-point scale Normal (7-8) Good (5-6) Fair (4-5) Poor (1-2)	71-76
	Speaking		
	Understanding		
	Reading		
	Other activities		
Functional assessment of communication skills (FACS)^39^	Social communication	Five-point scale Rating from “dose not” to “dose”	63,77,78
	Communication of basic needs		
		Seven-point scale: Rating from “never” to “always”	
	Reading, writing, and number concepts		
	Daily planning		

However, similar to other instruments typical of the functional approach, CADL is not linked to a particular theory of conversation; thus, performance cannot be interpreted with reference to the cognitive processes underlying communicative competence. Furthermore, CADL does not include formal definitions for coding and is affected by the influence of contextual variables such as familiarity with the topics of conversation and the type of relationship between interlocutors.^44^

***FCP***

FCP^38^^,^^45^ rates the effectiveness of communicative behavior in an informal conversation. FCP checklist consists of 45 items divided into five areas as movement, speaking, understanding, reading, and other activities. The use of a nine-point scale for rating FCP items leads to an overuse of intermediate-neutral category that, as a result, minimizes the likelihood of clear differences emerging.^42^ However, Sarno^38^ proposed a rather loose method of converting item grades into percentages that maybe too subjective for research purpose or for comparison with clinical evaluations made by different examiners.

***FACS***

FACS^39^ is an assessment tool available from American Speech-Language Hearing Association that scores 43 communicative abilities in four domains, which are social communication, communication of basic needs, reading, writing and number concepts, and daily planning. In comparison with FCP, FACS scores more precisely procedures and psychometric properties in daily life by using two scales for scoring; a seven-point scale for communication independence, which rates the level of assistance needed to complete a task, and a five-point scale for qualitative dimensions of communication, that rates adequacy, appropriateness, promptness and communication sharing. A possible shortcoming of FACS is that it is based on daily life of average, white Americans, however, adapted versions for other populations are being developed.

To better evaluate pragmatic skills, adherence to principles recommended in pragmatic and functional assessments seems essential. These include (i) evaluation in an open-daily like communication system, (ii) multidimensional assessment for characterization of language behaviors and cognitive processes underlying language, (iii) evaluation of individual’s adaptation, and (iv) examination of the effect of the pragmatic disorder on the person’s life. To summarize, pragmatics can be evaluated effectively in an interactional context by administration of the series of tasks requiring on-line processing, and then modification of context to observe adaptation.

## Conclusion

Pragmatics account for divergent aspects of communicative competence; those aligned with structure and those operate apart from the structural properties of utterances. Pragmatic impairments are not restricted to spoken language as pragmatics incorporates behaviors that encompass social, emotional, and communicative aspects of social interaction.^46^ In this regard, a proper pragmatic assessment can help speech pathologists studying different aspects of social and cognitive functioning that observation of non-verbal behaviors alone cannot, and also make a sound contribution to communication and social intervention strategies. As it was reviewed here, over the years many theories and measuring instruments have been developed for pragmatic abilities; different approaches have discrete perspectives on the definition of context and on the relative independence of pragmatic from other domains of language. Due to their variations, it is essential to first compare instruments’ features, such as level of scoring reproducibility and scope of analyzing components and then study pragmatics by desired tools. Authors hope that the current perspective provides basic information on the issue and can assist clinicians in selecting appropriate assessment approaches.

## Notes

**How to cite this article: **Sobhani Rad D. A review on adult pragmatic assessments. Iran J Neurol 2014; 13(3): 113-8.
